# Analysis of Bacterial Metabolites in Breath Gas of Critically Ill Patients for Diagnosis of Ventilator-Associated Pneumonia—A Proof of Concept Study

**DOI:** 10.3390/biom14121480

**Published:** 2024-11-21

**Authors:** Wojciech Filipiak, Robert Włodarski, Karolina Żuchowska, Alicja Tracewska, Magdalena Winiarek, Dawid Daszkiewicz, Marta Marszałek, Dagmara Depka, Tomasz Bogiel

**Affiliations:** 1 Department of Pharmacodynamics and Molecular Pharmacology, Faculty of Pharmacy, Collegium Medicum in Bydgoszcz, Nicolaus Copernicus University in Toruń, A. Jurasza 2 Str., 85-089 Bydgoszcz, Poland; 2 Department of Anaesthesiology and Intensive Care, 10th Military Research Hospital and Polyclinic, Powstańców Warszawy 5 Str., 85-681 Bydgoszcz, Poland; robert.wlodarski@10wsk.mil.pl; 3 Department of Microbiology, Faculty of Pharmacy, Collegium Medicum in Bydgoszcz, Nicolaus Copernicus University in Toruń, Maria Curie-Skłodowska 9 Str., 85-094 Bydgoszcz, Poland

**Keywords:** breath analysis, bacteria metabolites, ventilator-associated pneumonia (VAP), volatile biomarkers, diagnostic breath test, GC-MS metabolomic, *Acinetobacter baumannii*, *Escherichia coli*, *Klebsiella pneumoniae*, *Pseudomonas aeruginosa*

## Abstract

Bacterial infection of the lower respiratory tract frequently occurs in mechanically ventilated patients and may develop into life-threatening conditions. Yet, existing diagnostic methods have moderate sensitivity and specificity, which results in the overuse of broad-spectrum antibiotics administered prophylactically. This study aims to evaluate the suitability of volatile bacterial metabolites for the breath-based test, which is used for diagnosing Ventilator-Associated Pneumonia (VAP). The in vitro experiments with pathogenic bacteria most prevalent in VAP etiology (i.e., *Acinetobacter baumannii*, *Escherichia coli*, *Klebsiella pneumoniae*, and *Pseudomonas aeruginosa*) were performed to identify bacteria-derived metabolites using a specially designed cultivation system enabling headspace sampling for GC-MS analysis. Thirty-nine compounds were found to be significantly metabolized by tested species and, therefore, selected for monitoring in the exhaled breath of critically ill, mechanically ventilated (MV) patients. The emission of volatiles from medical respiratory devices was investigated to estimate the risk of spoiling breath results with exogenous pollutants. Bacterial metabolites were then evaluated to differentiate VAP patients from non-infected MV controls using Receiver Operating Characteristic (ROC) analysis, with AUC, sensitivity, and specificity calculated. Nine bacterial metabolites that passed verification through a non-parametric ANOVA test for significance and LASSO penalization were identified as key discriminators between VAP and non-VAP patients. The diagnostic model achieved an AUC of 0.893, with sensitivity and specificity values of 87% and 82.4%, respectively, being competitive with traditional methods. Further validation could solidify its clinical utility in critical care settings.

## 1. Introduction

Ventilator-Associated Pneumonia (VAP) is the most frequent infection acquired at the intensive care unit (ICU) that occurs mostly >48 h after endotracheal intubation necessary for mechanical ventilation [1]. VAP incidences vary widely from 5% to 40%, depending on ICU hygienic regimes, pathogen prevalence, and diagnostic criteria in local settings [1]. Patients suffering from VAP are at high risk of life-threatening complications, resulting in mortality exceeding 30% [2]. They also had significantly longer ICU and hospital length of stay, required longer time under invasive mechanical ventilation (MV), and were more often subjected to tracheostomy [3]. Considering the wide range of possible causative pathogens and severe complications if effective treatment is not induced timely, patients with suspected VAP commonly receive broad-spectrum antibiotics still before the diagnosis is confirmed, accounting for nearly half of all antibiotic use in the ICUs, which contributes to the escalation of antimicrobial resistance [4]. Moreover, despite available guidelines, different diagnosis criteria and management protocols exist across ICUs worldwide [5].

It was proven that applying different diagnostic criteria to the same patient population resulted in a delayed diagnosis (from 4 to 8 days) with increasingly stringent criteria, thus altering treatment and increasing mortality from 50% to 80% [6]. Also, the diagnostic methods lack specificity and sensitivity, whereby clinical examination, although essential, has a low sensitivity of 66% and specificity of 54% [6] and is nonspecific to VAP but often related to systemic inflammatory response. In turn, the presence of new or progressive infiltrates on chest X-ray (CXR) is a valuable adjunct for diagnosing VAP, offering as high sensitivity as 89%, but at the cost of specificity of only 26% (lung infiltrates on CXR imaging often overlap with other confounding pathologies, e.g., ARDS).

The microbiological examination is considered a gold standard in VAP diagnosis, but the variety of sampling techniques determines the limitations of this approach. For instance, although simple and safe, endotracheal aspirates cannot differentiate lower respiratory tract infection (LRTI) from colonization of upper airways [4]. Techniques that bypass upper airway colonization when collecting specimens from distal airways are bronchoalveolar lavage (BAL) and protected specimen brush (PSB) utilizing a fiberoptic bronchoscope. Both BAL and PSB techniques require an invasive procedure but yield good specificity (80% and 76%, respectively) and sensitivity (71% and 61%, respectively) [6]. To overcome the pitfalls of individual diagnostic methods mentioned above, a scoring system was proposed, where the clinical pulmonary infection score (CPIPS) is considered the most relevant for VAP diagnosis as it combines clinical, radiological, and microbiological criteria. The scores > 6 (for a scale ranging from 0 to 12) allow VAP diagnosis with a sensitivity of 74% and specificity of 66% [4,7]. Also, the biomarkers for VAP diagnosis are gaining increasing interest. Serum biomarkers like C-reactive protein (CRP) and procalcitonin are rather unspecific for VAP and, therefore, have shown similar to previous diagnostic accuracies of around 60–80% in distinguishing bacterial infections from uninfected controls, but these values vary between studies, making them inconsistent in diagnosing VAP [4].

Given the limitations of currently used methods, a pragmatic and non-invasive approach to VAP diagnosis is required that could indicate an emerging infection and reflect its progression or resolution within a short time (or real-time) [4]. These expectations seem to be met by another type of biomarkers recently investigated—the volatile organic compounds (VOCs), which were shown to be related to the pathological processes in patients suffering from diverse diseases [8,9,10,11,12,13]. Volatile biomarkers can be readily analyzed in the exhaled breath of critically ill patients in a non-invasive way, using mass spectrometry, either offline in the laboratory [14,15,16,17] or in real-time directly at the bedside of mechanically ventilated patients [18,19]. It was recently demonstrated that breath gas analysis is a valuable tool in diagnosing VAP with area under the curve (AUC) for Receiver Operating Characteristic (ROC) analysis ranging from 0.67 to 0.77 [20] in the case of individual VOCs, alternatively reaching AUC = 0.86 for a ROC model composed of 10 VOCs in another study [21]. However, a model breath test differentiating VAP from control patients included some compounds of unknown identity and other compounds of unknown or exogenous origin (e.g., enflurane).

We proposed another approach for arriving at a breath test for VAP diagnosis focusing on volatile metabolites with an unambiguously proven bacterial origin. Detection of such microbial metabolites in breath gas would testify to the presence of metabolically active bacteria, reflecting the ongoing infection. The increasing prevalence of antibiotic-resistant pathogens has been observed for decades [22], with a particular emphasis on the so-called ESKAPE group of microorganisms dominating the variety of hospital-acquired infections (HAI) [23]. Among them, the Gram-negative *Klebsiella pneumoniae*, *Acinetobacter baumannii*, *Pseudomonas aeruginosa*, and *Escherichia coli* are the most prevalent pathogens causing VAP [24,25]. Therefore, strains of these four species have been chosen for in vitro experiments investigating the production of volatile organic compounds.

This study aims to identify volatile compounds metabolized by these four bacteria, verify whether they can be detected in the breath gas of ventilated patients infected with those pathogens, and evaluate their diagnostic potential for VAP detection. To clarify the putative bias of incorporating exogenous compounds into a diagnostic model, the emission of volatiles from medical respiratory devices (endotracheal tube and disposable catheter mount) was additionally examined.

## 2. Materials and Methods

### 2.1. Chemicals

Gaseous and liquid chemicals (GC-MS standards) manufactured by Tokyo Chemical Industry (Tokyo, Japan), Sigma Aldrich (Merck KGaA, Darmstadt, Germany), Acros Organics, Alfa Aesar, and Honeywell (all three belong to Thermo Scientific Chemicals, Waltham, MA, USA) were purchased from AlChem (Torun, Poland).

### 2.2. Setup for Bacteria Cultivation and Headspace Sampling

*A. baumannii* (strain DSM 30008), *K. pneumoniae* (strain DSM 681), and *P. aeruginosa* (strain DSM 10273) were purchased from the German Collection of Microorganisms and Cell Cultures GmbH (Leibniz Institute DSMZ, Leibniz, Germany), while *E. coli* (strain ATCC 25922) was purchased from the American Type Culture Collection (ATCC, Manassas, VA, USA). Before the experiments, the strains were stored in tryptic soy broth (TSB, Becton Dickinson, Franklin Lakes, NJ, USA) with 15% glycerol at −80 °C.

An in-house system previously described in detail was used for bacteria cultivation and headspace gas sampling [26]. Briefly, glass bottles containing 100 mL of bacteria suspension in TSB (stirred with a rate of 80 rpm) were kept at 37 °C within a water bath, which was additionally placed inside an incubator at 45 °C to prevent water condensation in transfer lines that could lead to loss of volatiles. Synthetic air of purity 6.0 enriched with 5% CO_2_ (Air Products, Warsaw, Poland) was additionally purified with a Supelcarb filter (Merck KGaA, Darmstadt, Germany) and served as a carrier gas to transfer bacterial volatiles from cultures to sorption tubes filled with 140 mg of Carbotrap B (20/40 mesh) and 330 mg of Carbotrap X (60/80 mesh). The flow of carrier gas was split into two lines: (A) 5 mL/min passing the bacteria culture and (B) 40 mL/min used to dilute (hence decrease the humidity) the bacterial headspace. All the flows were precisely controlled with Mass Flow Controllers with the option of automatic flow compensation (Vögtlin Red-Y smart series, NewTech, Gliwice, Poland). As a quality control for the tightness of the entire headspace sampling system, the terminal flows at the outlet from sorption tubes during the sample extraction were also checked using a Mass Flow Meter (Vögtlin Red-Y Compact, NewTech, Gliwice, Poland). The volumes of 200 mL of headspace gas for GC-MS analysis were taken at 0 h (T0), 2 h 40 min (T1), 4 h 10 min (T2), 5 h 40 min (T3), 7 h 10 min (T4), 8 h 40 min (T5), 24 h (T6), and 26 h (T7) after inoculation of bacteria to a sterile TSB medium (except *A. baumannii*, which were discontinued after T5). Altogether, five biological replicates of each strain were measured in this study. After each headspace sampling, 300 µL of bacterial suspension was collected through a gas-tight septum port to assess bacterial growth and its quantification (CFU/mL).

### 2.3. VOCs Emission from Medical Respiratory Devices

The emission of volatiles from medical respiratory devices (MRD) was studied to verify whether the breath samples collected from mechanically ventilated patients were not contaminated with exogenous VOCs. For this purpose, the endotracheal tube cuffed model RIM-80 8.0 mm I.D. (ZARYS International Group, Zabrze, Poland) and the disposable catheter mount of the ventilator circuit model RB08-18-15 (Yangzhou Beswin Medical Equipment Co., Ltd., Yizheng Yangzhou, China), which were used at the ICU ward participating in this study, were examined. The experimental setup was as follows: Nitrogen 6.0 (Air Products, Warsaw, Poland), additionally purified on a Carboxen 1000 filter (Merck KGaA, Darmstadt, Germany), was used as a carrier gas passing (at a constant flow of 50 mL/min) through the examined respiratory devices, which were placed in an incubator kept at 37 °C (to mimic the temperature of a human body). The flow of nitrogen was initiated immediately after removal of both parts (endotracheal tubes and catheter mount) from their original sterile housing and their connection via short FEP capillary (5 cm length, 1/16” O.D., BOLA Bohlender GmbH, Grünsfeld, Germany) to the sorption tube of the same composition as used for breath sampling and in vitro experiments, i.e., 140 mg of Carbotrap B (20/40 mesh) and 330 mg of Carbotrap X (60/80 mesh). The total volume of 750 mL of nitrogen with the stripped-off contaminants released from medical respiratory devices was collected at 0, 1, 2, 3, 24, 25, 26, 27, 48, and 72 h counted since assembling the system and switching on the nitrogen flow. The same setup but without investigated medical devices was used to collect blank samples to estimate a systemic background for this test. The samples were measured on TD-GC-MS immediately after extraction on a sorption tube, using the same method as in vitro and in vivo experiments (bacteria cultures and breath gas, respectively).

### 2.4. Selection of Patients and Diagnosis of Pneumonia

All mechanically ventilated patients were recruited from the Anesthesiology and Intensive Care Unit of the 10th Military Research Hospital and Polyclinic in Bydgoszcz, Poland. Ethical approval for this study was obtained from the local Bioethics Committee (KB-218/2018). Patients who received mechanical ventilation for at least three days and developed VAP symptoms (i.e., with already confirmed VAP or at high risk) were eligible for breath sampling. The exclusion criteria were as follows: (1) age under 18; (2) pregnancy; (3) positive end-expiratory pressure (PEEP) >10; (4) increased intracranial pressure (ICP); (5) confirmed pre-existing SARS-CoV-2 infection and structural lung disease (traumatic lung injury or pulmonary cancer); (6) chest or lung injury; (7) extracorporal heart and lung assistance devices; and (8) strict isolation at the ICU. Ventilator settings such as ventilation mode, oxygen concentration in inspired air, and positive end-expiratory pressure (PEEP) were adjusted individually to prevent hypoxia or Ventilator-associated lung injury. An empirical antimicrobial therapy was introduced presumptively upon suspicion of pneumonia, considering pathogens’ local prevalence, resistance patterns, and patient factors (underlying disease, age, comorbidities, and immune status).

VAP was suspected based on radiology criteria: new and persisting infiltrates on chest X-ray or CT, assisted with the systemic signs: fever > 38 °C (with no other recognized cause), purulent secretion in the bronchial tree, auscultatory changes, and concomitantly elevated laboratory parameters: CRP > 50 mg/dL and PCT > 1 ng/mL. To confirm the clinical diagnosis of VAP, the positive result of microbiological culture from the BAL specimen was required at the quantity of >10^5^ CFU/mL with the coexistence of leucocytes in samples at the step of their microscopic evaluation.

The timing of breath sampling over the patient’s stay at the ICU was chosen to reflect potential changes in clinical status. Therefore, the period with the recognized progression of infection (based on clinical symptoms, inflammatory blood markers, and worsening respiratory parameters reflected with PaO_2_/FiO_2_ oxygenation and ventilator settings) was prioritized instead of a fixed day of mechanical ventilation. Patients with continuous VAP progression or resolution were eligible for additional collection of breath samples in subsequent days (following sampling day 1), except on weekends and in cases where activities belonging to the exclusion criteria were applied to the patient (most often: PEEP > 10, increased ICP, strict isolation, etc.). This approach allowed us to cover the broadest possible range of clinical conditions that could be observed in real-life ICU settings. In this manner, 77 breath samples were collected from thirty-two patients suffering from VAP, and 17 breath samples were collected from six uninfected patients receiving mechanical ventilation (i.e., from whom the collected BAL samples occurred to be culture-negative).

### 2.5. Collection of Breath Samples from Mechanically Ventilated Patients

All patients enrolled in this study were mechanically ventilated using a GE Healthcare CARESCAPE R860 ventilator (GE Medical Systems Polska Sp. z o.o., Warsaw, Poland) without an additional humidification system. The end-tidal air was collected directly from the respiratory circuit under continuous capnography control (i.e., visual monitoring of an exhaled CO_2_ profile) to maximize endogenous VOCs’ content and minimize exogenous VOCs’ contribution. Breath samples were collected once daily, at least 30 min after the last activity related to the patient (e.g., hygienic care). Each portion of exhaled gas (typically 20–40 mL, depending on the ventilation mode applied to the patient) was collected from the T-connector of the catheter mount of the ventilator circuit (Ref: RB08-18-15, Yangzhou Beswin Medical Equipment Co., Ltd., Yizheng Yangzhou, China), through a sterile bacterial filter (Ref: 7699822, pore size 0.20 μm, LabSolute, TH. Geyer GmbH, Renningen, Germany) to the glass syringe (250 mL total volume, Socorex Isba S.A., Ecublens, Switzerland). The glass syringes filled with 250 mL of breath gas samples were tightly closed and immediately placed into an incubator held at 45 °C for five to ten minutes to prevent water condensation and loss of polar analytes. Subsequently, a total volume of 750 mL of breath gas (i.e., three syringes) was extracted on a sorption tube (also thermostated at 45 °C) with a stable flow of 70 mL/min generated by a vacuum pump (Vacuubrand, Wertheim, Germany) and governed by a Mass Flow Controller (Vögtlin Red-Y smart series, NewTech, Gliwice, Poland). Immediately after extraction, sorption tubes were tightly sealed with Swagelok brass caps and transported from the Anesthesiology and Intensive Care Unit of the 10th Military Research Hospital to the Analytical Laboratory at the Department of Pharmacodynamics and Molecular Pharmacology of the Collegium Medicum NCU (both in Bydgoszcz, Poland), so the TD-GC-MS analyses were accomplished within several hours according to the protocol described below.

Notably, the respiratory conditions, such as oxygen content in the inspired air, the value of PEEP, alveolar recruitment, and ventilation mode, were adjusted to the individual needs of a patient without any relation to, and particularly not changed on the demand of, breath sampling. Therefore, the collection of exhaled air did not affect the patient’s condition, nor did it alter the composition of the collected breath sample (and results of GC-MS analysis). Similarly, bronchoalveolar lavage was performed only for microbiological testing upon suspicion of pneumonia (described above) and tailoring the antimicrobial therapy, and it was not related to the collection of breath samples from MV patients.

### 2.6. TD-GC-MS Analysis

One analytical protocol was used for TD-GC-MS analysis of all samples, including bacterial cultures, emissions from medical respiratory devices, and breath samples. Sorption tubes with extracted analytes were thermally desorbed at 320 °C over 15 min in a TD-30R autosampler (Shimadzu, ShimPol, Warsaw, Poland), and released VOCs were cryofocused at −20 °C on a cold trap filled with Carboxen. Rapid heating of the cold trap to 350 °C triggered splitless injection over 2 min into a Nexis 2030 Gas Chromatograph (Shimadzu, Shim-Pol, Warsaw, Poland). Sample constituents were separated on an Rt-Q-Bond capillary column 30 m × 0.25 mm × 8 µm (Restek, Bellefonte, PA, USA) using the following temperature program: initial 60 °C held for 2 min, ramp 8 °C/min to 110 °C (hold 1 min), ramp 3 °C/min to 120 °C (7 min), ramp 3 °C/min to 155 °C (7 min), ramp 3 °C/min to 225 °C (4 min), ramp 10 °C/min to 300 °C (7 min). Data were acquired with a QP-2020-NX Mass Spectrometer (Shimadzu, Shim-Pol, Warsaw, Poland) operating in a SCAN mode range of 33–235 *m*/*z*.

### 2.7. Data Processing and Statistical Analysis

Chromatographic data were processed according to the previously described protocol [26]. Shimadzu GC-MS PostRun Analysis software version 4.45 SP1 (combined with the NIST 2018 library) was used to identify and integrate peaks based on the predefined database of analytes. Notably, the experienced GC-MS analyst processing the data was blinded to the breath sampling procedure and any information related to the patient’s conditions, including clinical, microbiological, and laboratory results. To prevent errors in the integration of peak areas, the “Target Ion” was assigned to each analyte as the most selective ion (unique for only one compound in the case of coeluted peaks) or the most abundant ion (for peaks resolved to the baseline). Characteristic reference ions were also assigned to each analyte to verify peak identity during integration. Analytes were identified using the two-stage approach: by NIST 2018 spectra library match and by additional confirmation of retention times with injected standards. The integrated areas of all chromatographic peaks were pre-processed using Log10-transformation and Pareto scaling before performing the statistical tests using the Metaboanalyst 6.0 online software.

In the in vitro experiments, the significance between VOC levels in bacterial suspensions at different growth times and reference TSB medium was calculated using the Kruskal–Wallis test, which is a non-parametric test to compare samples from two or more groups of independent observations, whereby *p*-values < 0.05 were considered to be statistically significant. This test was selected because of its stability to outliers and no requirement for the groups to be normally distributed.

The bacterial metabolites elucidated in the in vitro experiments were then monitored in the breath gas collected from VAP and uninfected MV patients (culture-negative BAL). The significant *p*-values of the non-parametric Wilcoxon rank-sum test with correction to false discovery rate (FDR), as well as the values of Area Under the Curve (AUC) from Receiver Operating Characteristic (ROC) calculated with a Linear Supported Vector Machine algorithm (robust for features with low occurrence), and the high values of frequency in the Least Absolute Shrinkage and Selection Operator (LASSO) test were all considered to select individual compounds. Then, the AUC (calculated with the Random Forest algorithm, potentially capturing more complex non-linear relationships between the metabolites) and the True Positive Rate were used to evaluate the ROC model’s performance for chosen metabolite sets.

For the secondary aim of differentiating VAP patients based on the causative pathogen, a separate set of metabolites that potentially may be more pathogen-specific needed to be elucidated. For this reason, only patients with a single-pathogen infection were included in the model utilizing the Partial Least Squares Discriminant Analysis (PLS-DA). The metabolites with discrimination power between pathogen strains (based firstly on variable importance in projection, i.e., VIP scores, and secondarily on the statistical significance of the non-parametric one-way ANOVA test) were prioritized.

All statistical calculations and plotting were performed with Statistica version 13.3 PL software (TIBCO Software Inc., Tulsa, OK, USA) and the Metaboanalyst version 6.0 online software using Log10-transformation of chromatographic peak areas and Paretto-scaling.

## 3. Results

### 3.1. Bacteria Cultures

#### 3.1.1. Microbial Growth

**Figure 1:**
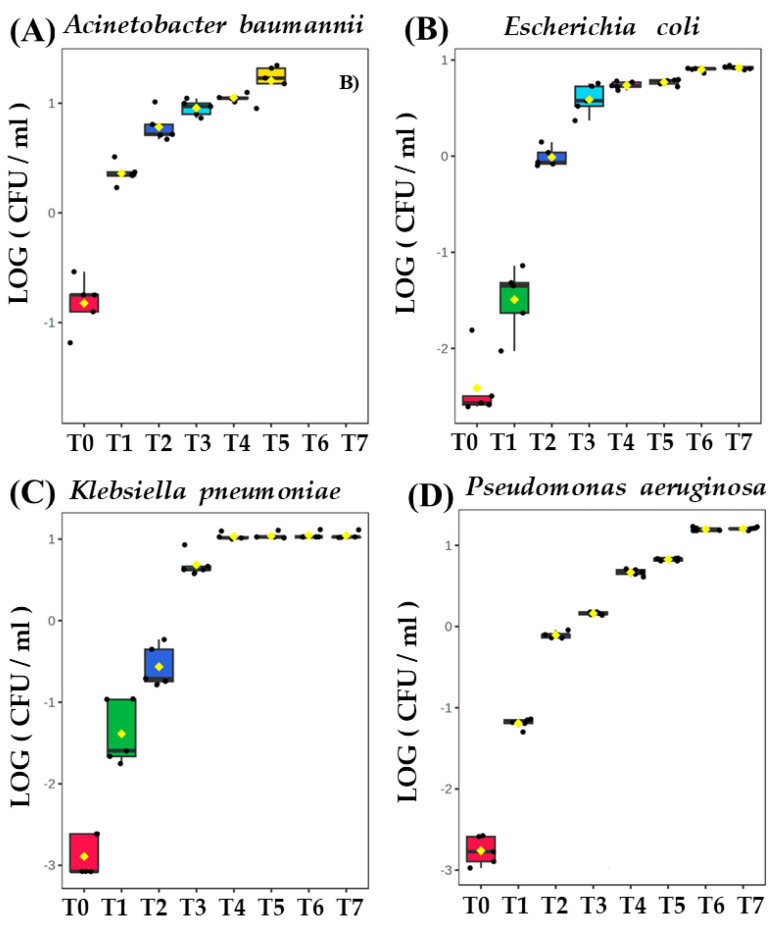
Growth curves of bacteria cultivated in vitro: (**A**) *Acinetobacter baumannii*, (**B**) *Escherichia coli*, (**C**) *Klebsiella pneumonia*, and (**D**) *Pseudomonas aeruginosa*. Colony-Forming Units (CFU/mL) are plotted after logarithmic transformation in the function of incubation time.

All bacterial strains were studied in five biological replicates. The initial bacteria density immediately after inoculation with TSB medium ranged from 4.0 × 10^4^ CFU/mL for *K. pneumoniae* to 1.7 × 10^6^ CFU/mL for *A. baumannii*. The latter exhibits the slowest proliferation rate and the lowest absolute cell density amongst all bacteria tested in this study, reaching only 3 × 10^8^ CFU/mL at T5 (8.6 h after inoculation), being two orders of magnitude less than for other species at the same time point (Supplementary Table S1). *E. coli*, *K. pneumoniae*, and *P. aeruginosa* exhibit nearly identical kinetics, whereby the cell density in experimental cultures ranged from approximately 1 × 10^5^ CFU/mL just after inoculation (T0) to approximately 3 × 10^10^ CFU/mL after 26 h (T7). The control measurements performed overnight (24 h for T6 and 26 h for T7) confirmed that the strains of these three bacterial species reached a steady state of growth, after which the experiment was discontinued (Figure 1).

#### 3.1.2. VOC of Microbial Origin

The primary aim of the performed in vitro experiments was to identify the volatile metabolites released by particular pathogenic bacteria. The secondary aim was to characterize the relation of their production kinetics to cultivation time or bacteria load.

The primary aim was approached by comparing the peak areas for respective metabolites analyzed at the same time points in the headspace of bacteria cultures and sterile reference medium. Using the non-parametric Kruskal–Wallis test, 39 VOCs were observed at significantly different levels in the headspace of bacteria cultures compared to the control medium. Amongst them, *A. baumannii* released fifteen VOCs and consumed another three substances from the broth medium. The relatively low number of compounds significant for this species was probably related to the slow metabolism of this bacteria, which is also reflected by its low proliferation rate. In turn, *E. coli* produced eighteen and utilized another seven metabolites. The most active metabolism of volatiles was observed for *K. pneumoniae*, whereby twenty-one VOCs were significantly released, and four were taken up by this species. On the contrary, only sparse production of VOCs was reported for *P. aeruginosa* strain DSM10273, whereby fourteen compounds were secreted and eight were taken up from the medium.

The adequately chosen time points for sampling bacterial headspace gas allowed us to cover the most dynamic phase of bacterial metabolism, namely the logarithmic phase of microbes proliferation. Consequently, three time-dependent profiles of VOC metabolism could be observed, including the release proportional to the bacteria load (RPB), release with a temporary maximum (RTM), after which VOC’s level decreases, and the uptake (UPT) of analyte from the surrounding medium (see Table 1 for examples).

**Figure 2:**
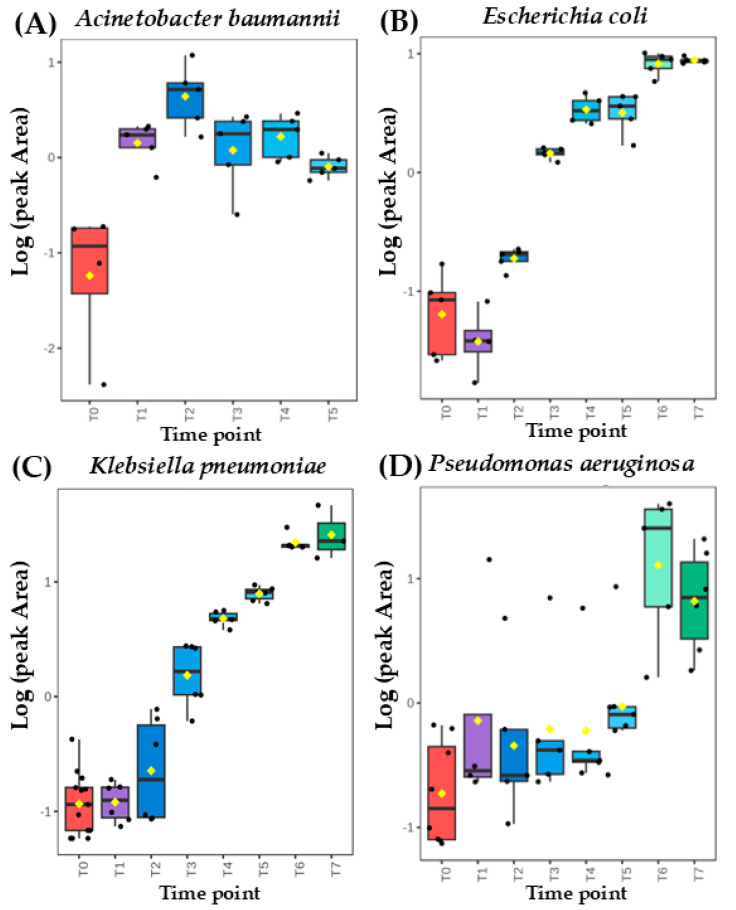
Comparison of time-dependent profiles for production of **ethyl acetate** from (**A**) *Acinetobacter baumannii*, (**B**) *Escherichia coli*, (**C**) *Klebsiella pneumoniae*, and (**D**) *Pseudomonas aeruginosa*.

**Figure 3:**
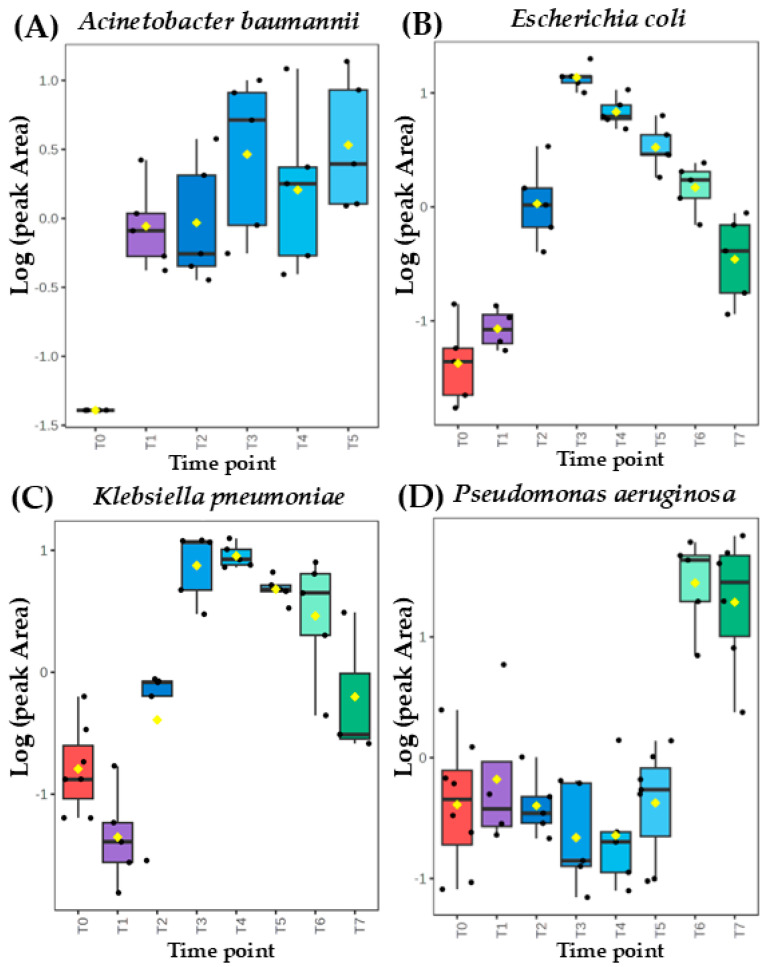
Comparison of time-dependent profiles for production of dimethyl sulfide from (**A**) *Acinetobacter baumannii*, (**B**) *Escherichia coli*, (**C**) *Klebsiella pneumoniae*, and (**D**) *Pseudomonas aeruginosa*.

Intrestingly, the same metabolite can exhibit a distinct secretion profile for different microorganisms. This can be well exemplified with ethyl acetate released with a temporary maximum by *A. baumannii* but in quantities proportional to the absolute bacteria load for the remaining three species (Figure 2). On the other hand, dimethyl sulfide’s abundance depended on the bacterial cell density for *A. baumannii* and *P. aeruginosa* but exhibits a clear and abundant temporary maximum for *K. pneumoniae* and *E. coli*. (Figure 3).

A closer look at the dynamically changing time profile allows us to find a few VOCs that exhibit even opposite profiles amongst bacteria, such as the case of 2-butene, which was produced by *A. baumannii* and *P. aeruginosa* but consumed by *E. coli*. A similar situation concerns aldehydes, where methacrolein and 2-methylpropanal were secreted by *A. baumannii* but taken up by all the remaining microorganisms studied, as well as 3-methyl butanal released by *K. pneumoniae* but degraded by *E. coli* and *P. aeruginosa* (Table 1).

### 3.2. Breath Analysis

#### 3.2.1. VOC Emissions from Medical Respiratory Devices

The most popular approach to elucidate biomarkers for certain diseases, including Ventilator-Associated Pneumonia, relies on untargeted analysis of collected samples to cover as broad a range of metabolites as possible with their subsequent reduction through adequately chosen statistical models. Apart from the problem with the correct identification of unknown analytes, there is also a problem with the appropriate processing of results obtained. Despite sound statistical models, incorrect substances, including exogenous contaminants or anesthetics, are often proposed as biomarkers. For this reason, the emission of volatiles from plastic materials constituting the respiratory circuit in mechanically ventilated patients placed near the infection site (endotracheal tube) or the breath sampling site (disposable catheter mount) was examined.

**Figure 4:**
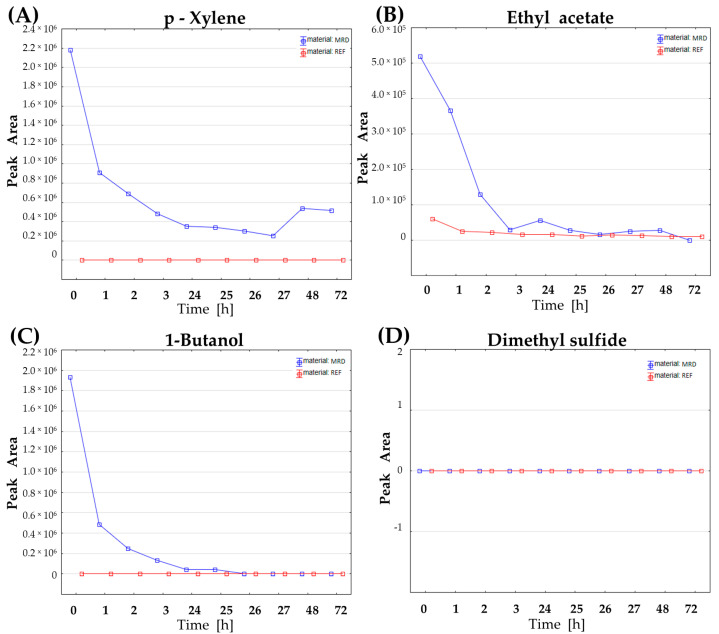
Emission of VOCs from the medical respiratory device parts (endotracheal tube with disposable catheter mount) for exemplary VOCs: (**A**) p-xylene, (**B**) ethyl acetate, (**C**) 1-butanol, and (**D**) dimethyl sulfide.

The results confirm that numerous substances of diverse chemical classes are released from the tested parts of a respiratory circuit (Supplementary Table S2). Amongst them, aromatic compounds (e.g., ethylbenzene, styrene, and xylene—Figure 4A) and heavier esters (containing more than six carbon atoms, >C6) do not reach the low level of reference blank samples within the tested time frame, suggesting a continuous emission of these impurities at a fixed level (Figure 4A). Importantly, also the low-molecular, very volatile compounds typically considered endogenous, including those with confirmed bacterial origin, e.g., ethyl acetate (Figure 4B), 2-methyl-1-propanol, and 1-butanol (Figure 4C), occurred to be secreted from MRD tested, but their levels decrease rapidly and reach nearly zero level within 24 h of a continuous rinse with nitrogen. Therefore, from a practical point of view, there is no risk of spoiling the results of breath analysis due to the emission of these endogenous VOCs from plastic materials if patients underwent mechanical ventilation for at least two or three days preceding collection of breath gas. Notably, the volatile sulfur-containing compounds (VSCs), such as dimethyl sulfide (Figure 4D) or dimethyl disulfide, which were proved to be of bacterial origin in the here presented and previous in vitro studies (thoroughly discussed elsewhere [13]), are not secreted from the tested MRD parts at all.

#### 3.2.2. Differentiation of VAP from Uninfected Patients

The most frequent reasons for admission to the intensive care unit in the studied population were cardiac (mostly cardiac arrest) and neurological (mainly stroke and intracerebral bleeding), less frequent organ failure (mostly pancreatitis and hepatic failure), or post-surgical complications (Table 2). Among injury cases, those concerning chest and lung structure were considered exclusion criteria.

The onset of VAP occurred on average on the 9th day of the patient’s mechanical ventilation, while the first breath sample was collected on average on the 7th day of ventilation in this group of patients. The most prevalent pathogens observed in this pilot study were *A. baumannii* (28%), followed by *P. aeruginosa* (19%). Both bacteria were also relatively often found to be the single causative pathogen, whereas the remaining microorganisms co-existed in most cases. The observed high mortality rate (83%) in the control group was the consequence of multi-organ failure not related to the bacterial infection, at least not localized in the lower respiratory tract. The inflammatory parameters differed substantially within the VAP cohort at the days of breath sampling, as exemplified by C-reactive protein reaching its maximum value of 18.54 mg/dL for a patient with *E. coli + K. pneumoniae + P. aeruginosa* strains coinfection and the maximum value of 497.96 mg/dL for a patient infected with a sole *P. aeruginosa* isolate. A similarly broad range of the results was observed for procalcitonin, reaching its maximum value of 0.06 ng/mL for a patient infected with *Staphylococcus aureus* and 177.06 ng/mL for a patient infected with *A. baumannii*. These data confirm the urgent need for reliable markers of bacterial infection in clinical settings.

Given the dynamics of inflammatory parameters and the likely change in pathogens causing VAP over a short time, the breath samples collected in multiple days capture distinct physiological conditions. On the one hand, this approach reflects different stages of the disease (e.g., early, mid, or late infection), providing meaningful information for the statistical model. On the other hand, changing conditions reduce the concern about sample duplication as well as bias related to patient-specific patterns and prevent sole sampling at the highest CRP/PCT values (which, as exemplified above, do not necessarily reflect the status of bacterial infection). Bearing this in mind, breath samples were collected from patients multiple times on different days if this did not disturb or interfere with routine clinical practice. Consequently, seventy-seven breath samples were collected from thirty-two VAP patients, and seventeen breath samples were measured from six control individuals whose microbiological cultures of collected BAL specimens remained culture-negative.

Only the in vitro confirmed metabolites (given in previous sections) were considered in the breath data analysis to ensure as high biological relevance of the results as possible. Amongst 39 VOCs significantly metabolized in vitro, 2-pentene was never found in the in vivo settings. The non-parametric Wilcoxon rank-sum test was performed for the remaining 38 VOCs detected in breath gas, revealing significantly different levels of 13 bacterial metabolites in breath samples collected from VAP and control non-infected individuals. Another two metabolites, dimethyl sulfide and methacrolein, exhibit p-values just slightly exceeding the threshold for significance (0.059 and 0.077, respectively). Given their low p-values and the production of both compounds by bacteria investigated in vitro, we decided to further verify the usefulness of these two substances in the breath model. The compounds with significant p-values from the Wilcoxon rank-sum test (plus dimethyl sulfide and methacrolein) were then arranged according to their AUC values, calculated for discrimination of VAP from control patients in ROC analysis. Next, the prevalence of a given compound in the breath samples was considered, prioritizing at least 50% in one of the compared groups of patients so the candidate for a biomarker could be broadly applicable. The value of 50% was not considered a cut-off threshold since the relatively low occurrence of metabolites in the group of VAP patients may be related to a particular pathogen and not subject to population-specific variations. In turn, to avoid the pitfalls of multicollinearity between compounds included in a ROC analysis, the LASSO modeling was used, whereby the frequency value of >70% was considered to indicate the compounds with strong predictive power (a reliable feature for the model), and the value of <30% suggests that the compound might be prone to noise or exhibit high collinearity with other features. Therefore, the LASSO frequency value equal to 30% was the least acceptable for including a compound in the ROC model, but on the condition that this substance has proven to have very high biological relevance. The number of bacteria species producing a particular compound is thus another factor respected in reducing candidate biomarkers to avoid the overfitting of the ROC model, prioritizing the metabolites released by multiple bacteria. The detailed parameters for each metabolite are given in Table 3.

**Figure 5:**
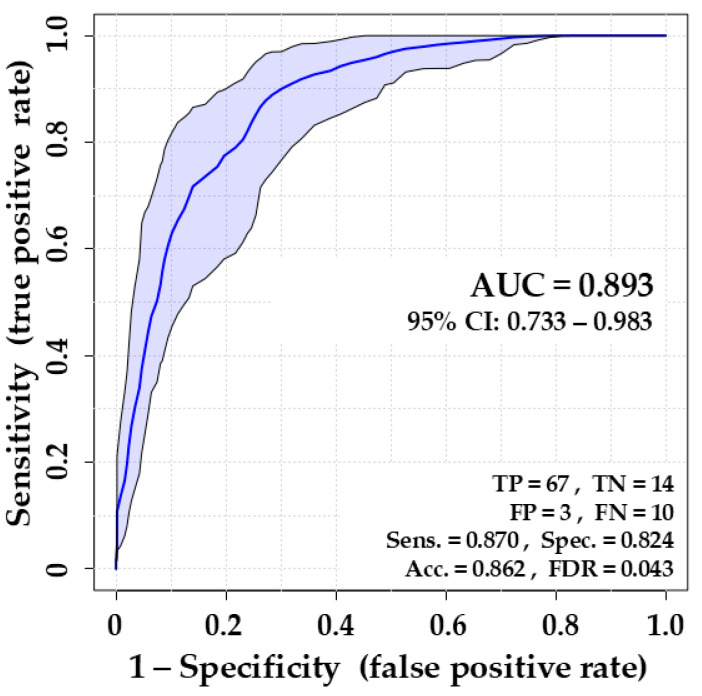
Performance of the Receiver Operating Characteristic (ROC) model composed of nine bacterial metabolites (ethyl acetate, 3-methyl-1-butanol, *n*-heptane, dimethyl disulfide, decanal, 1-butanol, ethyl methyl sulfide, dimethyl sulfide, and methacrolein) for discrimination of breath samples of VAP patients and uninfected controls. AUC: Area Under Curve; CI: Confidence Intervals; TP: True Positive; TN: True Negative; FP: False positive; FN: False Negative; Sens.: Sensitivity = TP/(TP+FN); Spec.: Specificity = TN/(TN + FP); Acc.: Accuracy = (TP + TN)/(P + N); FDR: False Discovery Rate = FP/(FP + TP).

According to the results of ROC analysis for individual features (Table 3), the following compounds with a significant *p*-value of <0.05 for the Wilcoxon rank-sum test do not reach the inclusion criteria for the multivariate model due to the LASSO penalization: 3-methyl-1-butene (frequency value of 0%), ethyl octanoate (10%), 1-octene (10%), 2-butene (20%), 2-nonanone (20%). Amongst the other three compounds reaching a required threshold LASSO frequency value of 30%, pentanal was excluded from further considerations as it was released by only one bacteria species (*E. coli*), whereas 1-butanol and dimethyl sulfide passed further as they were released by three and all four tested bacteria, respectively. Ultimately, the following nine compounds were included in the multivariate ROC model differentiating VAP and control individuals: ethyl acetate, 3-methyl-1-butanol, *n*-heptane, dimethyl disulfide, decanal, 1-butanol, ethyl methyl sulfide, dimethyl sulfide, and methacrolein. The performance of the ROC model composed of these nine bacterial metabolites is summarized in Figure 5.

The overall ability of the created ROC model based on nine bacterial metabolites to distinguish between positive (VAP) and negative (control) cases proved to be generally good, as the Area Under Curve reached the value of 0.893. The sensitivity (true positive rate) is 87%, allowing the correct identification of 67 out of 77 breath samples from VAP patients (leaving ten missed positive cases). It is an acceptable value, especially when supported with a solid specificity of 82% and an accuracy of 86%.

#### 3.2.3. Exhaled VOCs vs. Blood Biomarkers

The usability of currently monitored biomarkers (procalcitonin, C-reactive protein, and white blood cells) to differentiate VAP from control patients was investigated. To reveal whether the addition of these blood biomarkers to the exhaled VOCs will improve the performance of VAP diagnosis with a combined test, several classifications were performed. The sole blood-borne biomarkers occurred to be poor classifiers, reaching the AUC = 0.575 and accuracy = 0.649 for procalcitonin, AUC = 0.498 and accuracy = 0.596 for C-reactive protein, and AUC = 0.69 and accuracy = 0.681 for leukocytes. It was somehow expected that the lowest values for CRP would be observed, given that it is primarily a marker of inflammation of various causes and increases in such severe patients as those in the ICU, regardless of the bacterial infection.

Also, all three blood biomarkers together did not improve these parameters substantially, resulting in AUC = 0.707 and accuracy = 0.702. Combining procalcitonin, CRP, and leukocytes together with the selected nine bacterial VOCs in the breath yields nearly identical differentiation of VAP patients from non-VAP controls as with breath alone, reaching the AUC = 0.887, accuracy = 0.851. The detailed results of these comparisons are given in Supplementary Table S3. Notably, no correlation between exhaled volatile bacterial metabolites and blood-borne biomarkers could be found.

#### 3.2.4. VOCs Related to Underlying Pathogen

When focusing on the distinction between causative pathogens of VAP, one should assume that each species may have a unique metabolic signature or interact differently with the host. The volatile metabolites used to differentiate bacteria through exhaled breath analysis would need to reflect these pathogen-specific metabolic processes. For this purpose, it is reasonable to include the samples originating only from patients with a single-pathogen infection in the statistical calculation and exclude the cases with unknown contributions of co-existing mixed pathogens. Otherwise, instead of potentially unique volatile compounds indicating particular bacteria, a large set of VOCs released from all coexisting microorganisms would be found in patients with multi-pathogen infections that would not differ from each other. The putative inter-species interactions–which remain unknown–may further complicate VOC signatures, hindering the recognition of respective microorganisms. Splitting patients according to the causative pathogen results in a small number of breath samples within the sub-groups; hence, we do not rely solely on PLS-DA for selecting discriminating metabolites. Instead, the following approach was undertaken: First, the metabolites reaching high values of variable importance in projection (VIP-scores) from PLS-DA were identified. Then, the non-parametric univariate ANOVA was performed to confirm that these metabolites are statistically significant across pathogen groups.

Focusing only on the strains of four bacteria species investigated in this study, namely *A. baumannii*, *E. coli*, *K. pneumoniae*, and *P. aeruginosa*, altogether 13 VOC reaching VIP > 1 could be found, including dimethyl disulfide, 1-undecene, 2-propanol, dimethyl sulfide, 1-propanol, ethanol, benzaldehyde, isoprene, propanal, acetaldehyde, 2-butanone, 2,3-butanedione, and methanethiol (Supplementary Figure S1). The one-way non-parametric ANOVA (including correction for false discovery rate) proved the statistical significance for all of them except methanethiol (*p* = 0.05097). A more detailed comparison of significance for the exhaled metabolites mentioned above between different pathogen types was performed using the non-parametric Kruskal–Wallis test for multiple (two-sided) comparisons. The results revealed statistical significance for 11 out of 13 mentioned VOCs when compared between breath samples from patients infected with sole *A. baumannii* and sole *K. pneumoniae* (the only two non-significant for this pair of pathogens were ethanol and methanethiol). Six bacterial metabolites were found at significantly different levels in breath gas from VAP patients infected with *A. baumannii* and *E. coli* (dimethyl sulfide, isoprene, benzaldehyde, acetaldehyde, propanal, and 2,3/butanedione). The exhaled levels of three metabolites (1-undecene, dimethyl sulfide, and 1-propanol) reached statistical significance between patients infected with sole *K. pneumoniae* and sole *P. aeruginosa*. For the remaining comparisons, no statistically significant differences were noted (except ethanol for *A. baumannii* vs. *P. aeruginosa*). The p-values of pair-wise comparison by non-parametric Kruskal–Wallis test are listed in Supplementary Table S4.

Nevertheless, although the tested PLS-DA model passed the permutation test with *p* = 0.003 from 1000 simulations (which suggests that the model’s separation is better than random chance), it failed to pass the cross-validation, whereby both parameters evaluating the model were below the required value of 0.6, namely R^2^ = 0.384 (representing that the model does not fit well the training) and Q^2^ = 0.277 (reflecting the weak predictive ability of the model when applied to unseen data).

## 4. Discussion

Four microorganisms belonging to the most prevalent and dangerous pathogens in VAP patients were investigated in vitro regarding the metabolism of volatile organic compounds. The results confirm that two out of the reported 39 bacterial compounds, namely ethyl acetate and dimethyl sulfide, were produced by the strains of all four species, and the other nine compounds by three different pathogens, including methanethiol, ethyl methyl sulfide, dimethyl disulfide, acetaldehyde, isoprene, n-heptane, ethanol, 3-methyl-1-butanol, and 2-nonanone. On the contrary, four aldehydes, including methacrolein, 2-methylpropanal, 2-methyl-2-butenal, and benzaldehyde, were taken up by at least three pathogens under in vitro conditions. Consequently, the in vitro experiments presented here provide essential information in selecting bacteria-related volatiles that are highly potent for further in vivo studies.

The authors are aware that focusing only on 39 VOCs given in Table 1 in the analysis of breath samples collected from ventilated patients may strongly limit the significance of this study, but we aim to ensure as high biological relevance as possible in selecting the candidates for biomarkers for VAP diagnosis. Using metabolites with confirmed origin, which are produced by at least one of the most prevalent bacteria in ICU settings, should not only increase the probability of correctly classifying the VAP patient but also allow the detection of metabolically active bacteria that are involved in biological pathways relevant to disease onset or progression and not their inactive remnants (what is the risk of highly sensitive DNA-based analysis, such as PCR or LAMP).

The initially selected volatile metabolites of bacterial origin were carefully verified according to their values of AUC from ROC analysis, high LASSO frequency, significant *p* < 0.05 for a non-parametric ANOVA test with FDR correction, occurrence in breath samples, and prevalence in the in vitro studies with bacteria cultures. All these activities ensure that the metabolites chosen for the final ROC diagnostic model truly represent the active bacterial infection in the patients.

Concerning the here-applied approach of multiple sampling from the same patient, it is commonly accepted and even recommended that when considering the kinetics of biomarkers, the assessment of serial measurements over time is more informative than a single value [27]. As correctly pointed out by Miekisch and colleagues [28], single-point analyses are prone to erroneous interpretation (regardless of how sophisticated algorithms are applied to statistical analysis), especially when applied for primary diagnostic purposes. By covering the broader timeframe, breath samples collected from a patient over multiple (subsequent) days could capture the metabolite changes linked to the progression of the disease, improving the ROC diagnostic model to be more reflective of real-world clinical dynamics.

The performance of the presented diagnostic test is promising, especially considering the challenging nature of diagnosing VAP in critically ill patients due to the overlap of clinical symptoms and results of diagnostic methods with other common pathologies, e.g., such as pulmonary contusions, atelectasis, pulmonary edema, and particularly ARDS. Notably, exhaled volatile bacterial metabolites proved to be better classifiers than CRP, procalcitonin, and leukocytes considered individually or combined together. No further improvement in breath test performance after adding blood biomarkers indicates that the differentiation of patients with this combined classifier was based almost entirely on the detection of bacterial VOCs in the exhaled breath rather than on inflammatory parameters detected in blood. This stays in line with our observations in the current study where, in some cases, very high CRP (or PCT) levels were observed for patients in stable condition with VAP caused by a single pathogen and, on the other hand, low values of CRP and PCT in some patients with severe multi-pathogen infection (requiring re-adjustment of respiratory conditions and antimicrobial therapy). Both the results of patient classification based on combined blood-borne and exhaled biomarkers, as well as the observed weak relation of CRP and PCT to the severity of pneumonia in some cases, are in agreement with the results of other researchers, well reviewed by Grover et al. [29].

In this regard, the AUC of 0.893 indicates very good discriminative ability, differentiating between VAP and non-VAP patients in approximately 89% of cases. The AUC value reached in this study is higher than that reported for CPIS, ranging from 0.67 to 0.76 [7]. The AUC value of 89% is also superior to other recently reported studies on breath tests for VAP diagnosis [20,21].

A sensitivity of 87% is also very good, especially for life-threatening conditions like VAP, where missing a diagnosis could lead to severe consequences. Currently used clinical scores (CPIS) tend to have moderate sensitivity, often in the 60–80% range, depending on the population and specific criteria [7]. In turn, microbiological testing reaches sensitivity values of 71% for BAL and 61% for PSB. Compared to other breath studies focused on VAP diagnosis, the present sensitivity of 87% is lower than reported 98% [21] and 95% [20].

A specificity of 82% is comparable with those for microbiological testing of BAL (80%) and PSB (76%) [6] and higher than for CPIS score of 66% [7] or other breath-based test with 49% [21] and the range from 6% to 29% depending on individual substance [20].

An accuracy of 86.2% is generally considered good, indicating that the breath test correctly classifies patients in over 86% of cases. In the context of VAP diagnostics, an accuracy above 80% is typically regarded as strong, especially given the challenges in diagnosing VAP.

A false discovery rate (FDR) of 4.3% is a very good parameter, as it indicates a low proportion of false positives among the positive results. This reduces the likelihood of unnecessarily treating patients who do not have VAP, which is important in ICU settings where overuse of antibiotics can lead to escalation of antibiotic resistance and the development of multidrug-resistant pathogens. The evaluated performance of the proposed breath test, characterized by an AUC of 0.893, sensitivity of 0.870, and specificity of 0.824, suggests it is a promising diagnostic tool comparable to other methods used in ICU settings for VAP diagnosis.

Compared to the current standards of care, our research may bring potential benefits to patients in terms of a quick and non-invasive method of sample collection and instrumental analysis, which may translate into faster diagnosis than classic culture methods, allowing timely implementation of treatment. This, in turn, is a good prognostic for shortening the overall hospitalization time, reducing the risk of comorbidities and other side effects, and reducing ICU hospitalization costs (influencing an overall economic problem with the healthcare system). Nevertheless, authors feel the necessity to underline that any breath test—at the current stage of knowledge—should not replace nor compete with the existing diagnostic methods routinely used in clinical settings but complement the entire diagnostic procedure, particularly given the life-threatening nature of VAP and the high morbidity associated with missed diagnoses. Any claims for breath-based diagnostic models certainly need evaluation of dynamic changes in VOC profiles appearing not only in response to pathophysiological stimuli (such as emerging disease) but also to regular physiological/metabolic factors, as correctly pointed out by Kemnitz and colleagues [30]. Therefore, further validation in larger patient cohorts in multicenter studies and comparison with current standards (e.g., microbiological confirmation) would be necessary to fully assess its clinical applicability.

## 5. Challenges and Study Limitations

The conceptual challenge of this study is the nature of breath analysis in general, as the concentrations of exhaled VOCs detected in the breath of mechanically ventilated patients can be affected by several factors, including ventilation settings and patient physiology. The influence of those parameters still needs to be evaluated to ensure reproducibility and manage variability, which is crucial to estimating the robustness of the proposed breath test.

Although focusing on VOCs with proven bacterial origin, identifying the specific microorganisms causing VAP by analyzing exhaled VOCs still requires further refinement, as the model presented here failed. Most probably, the small population size of patients enrolled in this study (when divided respectively by underlying pathogen) was insufficient to approach this problem accurately, indicating the main limitation of this study. Given the number of pathogens causing VAP and their potential interactions determining VOC metabolism [26], detecting pathogen-specific breath markers in clinical settings will be a formidable task, if realistic at all. Nevertheless, the indication of bacterial lung infection achieved in this study, although without identifying the underlying pathogen, is a significant clinical advantage, as it intensifies clinicians’ efforts for the particular patient. Moreover, collecting exhaled air could also be used when, for some reason, BAL cannot be taken from the patient for anatomical, post-traumatic, functional, clinical reasons, etc., or when it is risky for the above-mentioned reasons and should be abandoned, because these may be clear contraindications to any invasive procedure.

## 6. Conclusions

The non-invasive nature of monitoring VOCs in breath gas and the unlimited accessibility of these samples are the obvious advantages over the currently used BAL sampling for its culture in VAP diagnosis. Typically, non-invasive methods like clinical scoring systems or biomarkers (e.g., procalcitonin) often exhibit a moderate level of performance (sensitivity and accuracy ranging from 70 to 80%). The presented diagnostic breath-based test is competitive with most existing clinical methods for diagnosing VAP, reaching performance at 80–90% (AUC = 0.893, sensitivity = 0.870, and specificity = 0.824). Notably, only the volatile compounds linked to bacterial metabolism, reflecting the pathogen’s presence and ongoing infection, are directly targeted in the proposed breath-based test, improving its biological relevance. Altogether, the gathered results suggest this test might be a promising tool for VAP diagnosis, where high sensitivity is particularly important to prevent the development of life-threatening lung infections associated with high morbidity. However, further validation in larger patient cohorts in multicenter studies and comparison with current standards (e.g., microbiological confirmation) would be necessary to solidify its clinical utility in critical care settings.

## Figures and Tables

**Table 1:** VOCs metabolized by *Acinetobacter baumannii* (AB), *Escherichia coli* (EC), *Klebsiella pneumoniae* (KP), and *Pseudomonas aeruginosa* (PA). The upward arrow (↑) indicates the release of a respective metabolite either with the amount proportional to the bacteria load (RPB) or release with the temporary maximum (RTM), after which the abundance of metabolites decreases. The downward arrow (↓) indicates an uptake (UPT) of a respective metabolite from the TSB medium. CAS stands for Chemical Abstract Service, providing a unique ID number for each substance.

VOCs	CAS	t_R_ [min]	Target Ion [*m*/*z*]	Reference Ions [*m*/*z*]	AB	EC	KP	PA
2-Butene	107-01-7	12.766	41	56, 39, 55	↑RTM	↓UPT	-	↑RPB
Isoprene	78-79-5	24.927	67	68, 53, 39	↑RPB	↑RTM	↑RTM	-
3-methyl-1-butene	563-45-1	24.148	55	70, 41, 42	-	-	↑RPB	↑RPB
(E)-2-Pentene	109-68-2	25.680	55	70, 53, 56	↑RPB	-	↑RTM	-
n-Heptane	142-82-5	51.257	71	57, 43, 100	↑RTM	↑RTM	-	↑RPB
1-Octene	111-66-0	58.528	55	70, 41, 83	-	↑RPB	-	-
1-Undecene	821-95-4	73.600	55	70, 83, 97, 111	↑RPB	↑RPB	-	↑RPB
Acetaldehyde	75-07-0	9.644	43	44, 42, 41	↑RTM	↑RPB	↑RPB	-
Propanal	123-38-6	18.263	57	55, 39, 37	↑RTM	↑RPB	-	-
Methacrolein	78-85-3	28.261	70	41, 39, 42	↑RTM	↓UPT	↓UPT	↓UPT
2-Methylpropanal	78-84-2	28.588	72	41, 43, 39	↑RTM	↓UPT	↓UPT	↓UPT
Butanal	123-72-8	31.169	44	72, 57, 41	-	-	-	↓UPT
2-Butenal	4170-30-3	35.863	70	41, 39, 69	-	↑RTM	-	-
2-Methyl-2-Butenal	1115-11-3	47.964	84	55, 39, 41	-	↓UPT	↓UPT	↓UPT
2-Ethylacrolein	922-63-4	41.552	55	84, 56, 39	-	↓UPT	-	↓UPT
3-Methylbutanal	590-86-3	42.417	58	71, 41, 86	-	↓UPT	↑RTM	↓UPT
Pentanal	110-62-3	45.598	44	58, 41, 57	↓UPT	-	-	-
Benzaldehyde	100-52-7	65.584	106	105, 77, 51	↓UPT	↓UPT	↓UPT	↓UPT
Octanal	124-13-0	70.320	84	69, 56, 100	↓UPT	-	-	-
Decanal	112-31-2	76.452	57	43, 55, 82	-	↑RTM	-	-
Ethanol	64-17-5	14.000	45	46, 43	-	↑RPB	↑RPB	↑RPB
1-Propanol	71-23-8	26.439	59	42, 60, 41	-	↑RPB	↑RPB	-
2-Propanol	67-63-0	22.209	45	43, 41, 59	-	-	-	↑RPB
2-Methyl-1-Propanol	78-83-1	36.500	43	41, 56, 74	-	↑RPB	↑RPB	-
1-Butanol	71-36-3	39.800	56	45, 41, 43	↑RTM	-	↑RTM	-
3-Methyl-1-Butanol	123-51-3	50.395	55	70, 42, 41	↑RPB	↑RPB	↑RPB	-
Ethyl Acetate	141-78-6	33.382	43	70, 61, 45, 88	↑RTM	↑RPB	↑RPB	↑RPB
Ethyl Octanoate	106-32-1	75.589	88	57, 101, 127	-	↑RTM	↑RTM	-
2-Butanone	78-93-3	31.539	72	43, 57, 42	-	-	↑RPB	-
2,3-Butanedione	431-03-8	31.663	86	43, 42, 41	-	-	↑RTM	-
3-Penten-2-on	625-33-2	48.521	69	84, 55, 41	-	↑RTM	-	-
2-Pentanone	107-87-9	44.911	43	86, 71, 58	-	-	↑RPB	↑RPB
2-Heptanone	110-43-0	63.400	58	43, 71, 114	-	-	↑RPB	↑RTM
2-Nonanone	821-55-6	73.080	58	43, 71, 142	↑RPB	-	↑RPB	↑RPB
Methanethiol	74-93-1	10.900	47	48, 45, 46	↑RTM	-	↑RPB	↑RTM
Dimethyl Sulfide	75-18-3	20.432	62	47, 45, 61	↑RPB	↑RTM	↑RTM	↑RPB
Ethyl Methyl Sulfide	624-89-5	32.634	61	76, 48, 47	-	↑RTM	↑RTM	↑RPB
Dimethyl Disulfide	624-92-0	47.136	94	43, 79, 61	-	↑RPB	↑RPB	↑RPB
2-Methylfuran	534-22-5	30.814	82	53, 81, 39	-	-	-	↓UPT

**Table 2:** Demographic characteristics of patients with confirmed Ventilator-Associated Pneumonia (VAP) and control individuals in whom VAP was excluded based on culture-negative results from BAL specimens. APACHE II—Acute Physiology and Chronic Health Evaluation score; IQR—Interquartile Range; * BAL culture results—may concern multiple pathogens detected.

		Control (n = 6)	VAP (n = 32)
**Age, years**	**median (IQR)**	80 (64–86)	68 (56–75)
**Male**	**n (%)**	5 (83)	19 (57)
**Admission Type**	**cardiac**	2	10
	**neurological**	2	5
	**organ failure**	0	4
	**post-surgical**	0	4
	**injury**	1	2
	**other**	1	7
**APACHE II**	**median (IQR)**	37 (27–40)	30 (27–33)
**Days from intubation to VAP diagnosis**	**median (IQR)**	not concerns	7 (4–18)
**Ventilation days at the 1st breath sampling**	**median (IQR)**	6 (3–14)	9 (6–19)
**ICU days**	**median (IQR)**	15 (8–32)	25 (14–33)
**ICU mortality**	**n (%)**	5 (83%)	16 (48%)
* BAL culture results	**n (single)**		
***Acinetobacter baumannii***		-	16 (11)
***Pseudomonas aeruginosa***		-	11 (6)
***Escherichia coli***		-	5 (1)
***Klebsiella pneumoniae***		-	4 (2)
***Staphylococcus aureus***		-	6 (3)
***Enterococcus aerogenes***		-	5 (1)
***Proteus mirabilis***		-	3 (0)
**Other**		-	8 (0)

**Table 3:** Results of ROC analysis discriminating breath samples collected from VAP (n = 77) and control (n = 17) individuals. AUC was calculated using the Linear Supported Vector Machine algorithm, which is robust for compounds with low occurrence in the tested population. For VOCs with statistical significance (*p* < 0.05 in Wilcoxon Rank-Sum test), the Lasso frequency values >70 (indicating strong predictive power) are given in bold, and <30 (indicating collinearity or noise) are given in red font.

VOCs	VAP n > 0	VAP	Control n > 0	Control	Wilcoxon Rank-Sum	LASSO Freq. [%]	AUC
Ethyl Acetate	77	100%	14	82%	0.00001	**100**	0.824
3-Methyl-1-Butanol	51	66%	3	18%	0.00010	**100**	0.762
*n*-Heptane	75	97%	13	76%	0.00014	**100**	0.753
Dimethyl Disulfide	25	32%	0	0%	0.00740	**100**	0.573
1-Octene	75	97%	13	76%	0.00093	10	0.568
Decanal	49	64%	3	18%	0.00638	**100**	0.56
Pentanal	54	70%	6	35%	0.01730	30	0.533
1-Butanol	19	25%	0	0%	0.02416	30	0.492
2-Nonanone	3	4%	3	18%	0.04134	20	0.488
2-Butene	77	100%	17	100%	0.02641	20	0.486
Ethyl Methyl Sulfide	0	0%	4	24%	0.00002	**80**	0.467
3-methyl-1-butene	76	99%	17	100%	0.01744	0	0.467
Ethyl Octanoate	0	0%	3	18%	0.00021	10	0.459
Dimethyl Sulfide	75	97%	14	82%	0.05927	30	0.452
Methacrolein	77	100%	16	94%	0.07703	**90**	0.432
2,3-Butanedione	75	97%	12	71%	0.12534	50	0.434
3-Methylbutanal	24	31%	7	41%	0.13259	60	0.466
2-Butenal	20	26%	7	41%	0.14670	90	0.448
2-Heptanone	10	13%	4	24%	0.16042	60	0.466
Benzaldehyde	77	100%	13	76%	0.17835	40	0.432
2-Methylpropanal	50	65%	5	29%	0.21740	100	0.475
2-Propanol	71	92%	17	100%	0.23453	100	0.437
2-Butanone	77	100%	16	94%	0.32112	10	0.447
3-Penten-2-on	45	58%	6	35%	0.32642	0	0.55
2-Methyl-2-Butenal	8	10%	3	18%	0.34197	10	0.480
2-Ethylacrolein	12	16%	1	6%	0.34245	100	0.498
Methanethiol	70	91%	14	82%	0.34789	60	0.493
Isoprene	77	100%	17	100%	0.38197	100	0.485
1-Undecene	40	52%	9	53%	0.41677	20	0.481
2-Methyl-1-Propanol	9	12%	1	6%	0.44076	40	0.492
2-Methylfuran	77	100%	17	100%	0.46127	0	0.476
Ethanol	73	95%	17	100%	0.53599	40	0.511
Acetaldehyde	77	100%	17	100%	0.70894	0	0.514
2-Pentanone	74	96%	17	100%	0.71625	70	0.518
Octanal	55	71%	9	53%	0.76448	100	0.531
1-Propanol	56	73%	17	100%	0.95274	80	0.507
Propanal	75	97%	16	94%	0.96866	90	0.522
Butanal	77	100%	16	94%	1.00000	10	0.553

## Data Availability

The dataset used and analyzed during the current study is available from the corresponding author upon reasonable request.

## Supplementary Materials

The following supporting information can be downloaded at: https://www.mdpi.com/article/10.3390/biom14121480/s1, Table S1: proliferation rate of bacteria grown in vitro. Table S2: VOC emissions from medical respiratory devices. Table S3: ROC variants with clinical parameters. Table S4: Non-parametric Kruskal–Wallis test for significance of exhaled VOCs in patients infected with different pathogens. Figure S1: VIP scores for compounds included in the PLS-DA model differentiation pathogen types by breath gas analysis.
