# Supplementary figures and images for: Analysis of Bacterial Metabolites in Breath Gas of Critically Ill Patients for Diagnosis of Ventilator-Associated Pneumonia—A Proof of Concept Study
Source: Biomolecules. 2024 Nov 21;14(12):1480. doi: 10.3390/biom14121480 (PMC11727074; doi:10.3390/biom14121480)

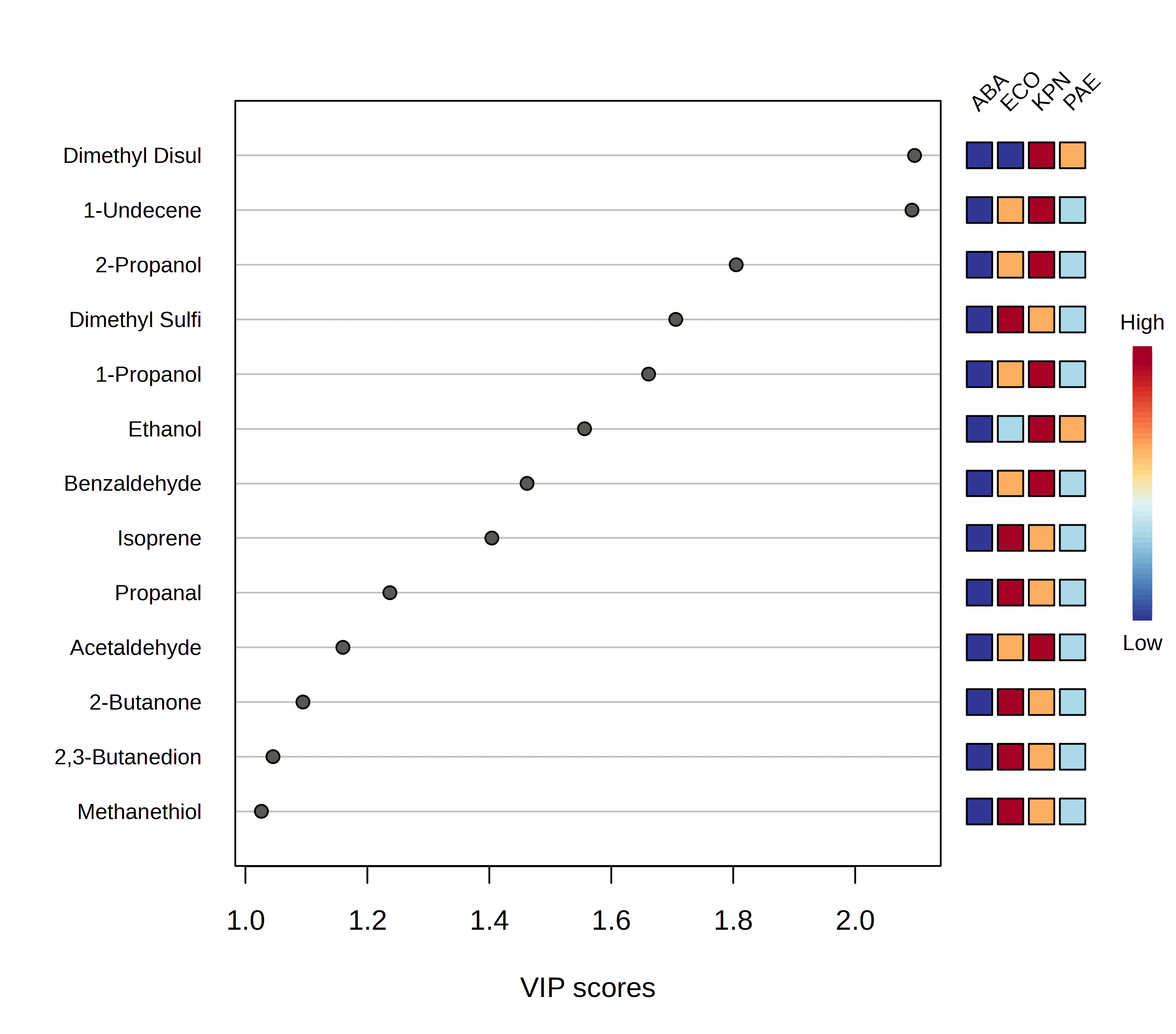

Supplement: Supplementary file 1 [file biomolecules-14-01480-s001.zip › Supplementary Figure SF1 - VIP_score__for__PLSDA-MULTIpathogen.png]
